# Osteoporosis Is Associated with Cerebral Small Vessel Disease in Stroke-Free Individuals: A Retrospective Observational Study

**DOI:** 10.3390/geriatrics10030066

**Published:** 2025-05-09

**Authors:** Xueling Xiao, Luling Chen, Manxiang Deng, Jingqi Liu, Jiayan Cai, Chuhan Su

**Affiliations:** 1 Department of Geriatrics, Zhongshan Hospital Xiamen University, Xiamen 361004, China; xiaoxueling@xmzsh.com (X.X.);; 2 Department of Radiology, Zhongshan Hospital Xiamen University, Xiamen 361004, China

**Keywords:** cerebral small vessel disease, neuroimaging markers, bone mineral density, osteoporosis, osteopenia

## Abstract

**Objectives**: This study aimed to investigate the relationship between osteoporosis and cerebral small vessel disease (CSVD) burden in stroke-free individuals, as well as its specific imaging markers, including lacunes, enlarged perivascular spaces (EPVSs), white matter hyperintensities (WMHs), and brain atrophy (BA). **Methods**: A total of 684 stroke-free patients who underwent both bone mineral density (BMD) assessments and brain MRI were included. Clinical data, CSVD burden scores, imaging markers of CSVD, and bone density parameters were collected. Logistic regression models were used to evaluate the relationship between BMD and CSVD burden and imaging markers. **Results**: Osteoporosis, including hip and vertebral osteoporosis, was independently associated with CSVD burden (OR = 2.332, 95%CI: [1.345, 4.039], *p* = 0.003; OR = 2.598, 95%CI: [1.540, 4.384], *p* < 0.001; OR = 1.515, 95%CI: [1.010, 2.272], *p* = 0.044). Increased BMD in the hip and spine correlated with reduced CSVD burden (OR = 0.929, 95%CI: [0.887, 0.972], *p* = 0.001; OR = 0.952, 95%CI: [0.917, 0.989], *p* = 0.012). Hip osteoporosis was a risk factor for lacunes (OR = 2.215, 95%CI: [1.197, 4.1], *p* = 0.011), multiple lacunes (OR = 2.274, 95%CI: [1.039, 4.980], *p* = 0.04), severe WMH (OR = 2.611, 95%CI: [1.171, 5.823], *p* = 0.019), and EPVS ≥ 2 (OR = 1.99, 95%CI: [1.133, 3.495], *p* = 0.017). No significant association was found between osteoporosis and BA (*p* = 0.928). In sex-stratified analyses, both hip and vertebral osteoporosis were independently associated with a higher CSVD burden in female patients (hip: OR = 2.529, 95%CI: [1.122, 5.703], *p* = 0.025; vertebral: OR = 3.129, 95%CI: [1.517, 6.455], *p* = 0.002; general osteoporosis: OR = 1.755, 95%CI: [1.057, 2.912], *p* = 0.03), whereas no significant association was observed in male patients (all *p* > 0.05). **Conclusions**: Osteoporosis was independently associated with an increased burden of CSVD, particularly evident in female patients. These findings suggest that bone health may be important in CSVD management, particularly for women.

## 1. Introduction

Cerebral small vessel disease (CSVD) is a group of disorders marked by abnormalities in the cerebral small vessels. Characteristic magnetic resonance imaging (MRI) features are used to define CSVD, including lacunes, enlarged perivascular spaces (EPVSs), cerebral microbleeds (CMBs), white matter hyperintensities (WMHs), and brain atrophy (BA). CSVD causes about 25% of ischemic strokes, most vascular dementia, and most intracerebral hemorrhages in the elderly over 65, and is associated with motor impairment, gait apraxia, neurobehavioral abnormalities, and mood disorders [1]. Previous research has identified a range of conventional vascular risk factors for CSVD [2]. However, despite extensive studies in this area, the biological mechanisms underlying CSVD remain insufficiently understood [3,4,5]. Thus, identifying new biomarkers could significantly enhance our understanding of CSVD, laying the groundwork for advances in clinical management.

Osteoporosis, a systemic bone disease characterized by low bone mass and deterioration of bone tissue microarchitecture, leading to increased bone fragility and a higher risk of fractures [6], is linked to cardiovascular diseases [7,8,9,10], including coronary artery disease and carotid atherosclerosis. Moreover, evidence suggests that osteoporosis is related to neurological disorders [11,12,13,14,15], such as Alzheimer’s and Parkinson’s disease. Therefore, we hypothesized that osteoporosis might be associated with CSVD. Only a few studies have explored the relationship between osteoporosis and CSVD. Existing studies have primarily focused on stroke populations [16] or examined only specific CSVD features in healthy individuals. Research in non-stroke populations has linked low bone mineral density to various brain structural changes, including gray matter atrophy [17], brain parenchymal atrophy [18], ventricular enlargement [19], and white matter hyperintensities [20]. However, there is currently a lack of comprehensive studies investigating the overall association between osteoporosis and CSVD burden in stroke-free individuals. Given the importance of early prevention, our study aims to fill this gap by systematically assessing the relationship between osteoporosis and both total CSVD burden and its specific imaging markers. We aim to provide new evidence on the connection between osteoporosis and CSVD. Understanding this link will help us gain a more comprehensive understanding of the pathophysiology of these diseases, ultimately leading to more effective prevention and treatment approaches.

## 2. Materials and Methods

### 2.1. Study Participants and Data Collection

**Figure 1:**
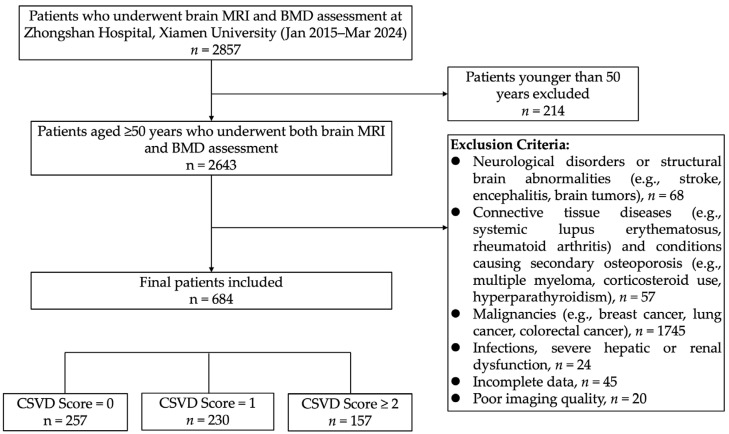
Flowchart of participant selection for this study.

We retrospectively included patients aged 50 years and older who underwent both brain MRI and BMD assessments at Zhongshan Hospital Affiliated to Xiamen University between 2015 and 2024. Exclusion criteria were as follows: (1) neurological disorders or structural brain abnormalities (e.g., acute or prior cerebral infarction, cerebral hemorrhage, traumatic brain injury, intracranial mass lesions, history of brain surgery, encephalitis, cerebral vasculitis, cerebral vascular malformations, or multiple sclerosis); (2) connective tissue diseases or conditions associated with secondary osteoporosis (e.g., systemic lupus erythematosus, rheumatoid arthritis, multiple myeloma, primary hyperparathyroidism, or long-term corticosteroid use); (3) malignancies (e.g., breast, lung, or colorectal cancer, or other active cancers); (4) severe comorbidities or systemic conditions (e.g., severe hepatic or renal insufficiency, recent infections, or other serious illnesses); (5) poor imaging quality; and (6) incomplete data. In total, 684 patients were ultimately included, as illustrated in the patient selection flowchart (Figure 1). The study population primarily consisted of (1) individuals undergoing routine health check-ups, who opted for both tests as part of a comprehensive evaluation of bone and cerebrovascular health; (2) patients with symptoms such as lower back pain, dizziness, or balance issues, prompting DXA or brain MRI for further assessment; and (3) hospitalized or outpatient individuals for whom physicians recommended both examinations due to risk factors like advanced age, hypertension, or diabetes.

We collected baseline data from the Hospital Information System, including demographic characteristics, medical history (diabetes, hypertension, dyslipidemia, coronary heart disease (CHD), atrial fibrillation (AF), peripheral artery disease (PAD), hyperuricemia), blood laboratory parameters (white blood cell count (WBC), red blood cell count (RBC), hemoglobin (Hb), low-density lipoprotein cholesterol (LDL-C), triglycerides (TGs), total cholesterol (TC), fasting plasma glucose (FPG), alanine aminotransferase (ALT), estimated glomerular filtration rate (eGFR), C-reactive protein (CRP), D dimer (DD), total protein (TP), albumin (Alb), fibrinogen (Fbg), etc.), current smoking and alcohol consumption status, body mass index (BMI), and systolic and diastolic blood pressures (SBP and DBP).

### 2.2. Measurement of CSVD

Brain MRI sequences included T1-weighted images (T1WI), T2-weighted images (T2WI), fluid-attenuated inversion recovery (FLAIR), diffusion-weighted imaging (DWI), and susceptibility-weighted imaging (SWI). The field of view (FOV) was 240 mm × 240 mm, with a matrix size of 320 × 256. Coronal and transaxial views of T1WI and T2WI and transaxial views of T2-FLAIR, DWI, and SWI were collected. All MRI data were obtained from the Picture Archiving and Communication Systems and independently reviewed by an experienced chief radiologist and a senior neurologist with extensive experience in cerebrovascular diseases. Both evaluators were blinded to the clinical information of the subjects. Any discrepancies in interpretation were resolved through discussion and consensus.

All neuroimaging markers of CSVD, including lacunes, WMH, EPVS, CMBs, and BA, were strictly diagnosed according to the Standards for Reporting Vascular Changes on Neuroimaging (STRIVE) guidelines [21,22]. Lacunes were defined as round or oval lesions of cerebrospinal fluid signal on T1WI and T2WI measuring 3 to 20 mm in diameter, always with a hyperintense ring on FLAIR. WMH was defined as abnormal signals of varying sizes in periventricular and deep white matter regions, which were hyperintense on T2WI and FLAIR and isointense or hypointense on T1WI. The severity of periventricular (PVWMH) and deep WMH (DWMH) were assessed according to the Fazekas scale from 0 to 3 [23]. EPVS appeared as small (<3 mm) punctate or linear structures with CSF signal intensity on T1WI, T2WI, and FLAIR in the centrum semiovale (CS-EPVS) and basal ganglia (BG-EPVS). The severity of EPVS in both regions was assessed separately by a previously validated grading system (0–4) [24], and the final grade was determined by the worse of the two. CMBs were defined as round lesions less than 10 mm in diameter with hypointensity on SWI. A visual cortical atrophy rating scale (0–3) was used to assess whole-brain cortical atrophy. Due to the low number of participants rated as Grade 3 (only 4 cases) in this study, these were combined with Grade 2 for analysis.

Finally, the total burden score of CSVD was assessed according to the STRIVE-1 criteria: 1 point was assigned for a DWMH score of 2 or a PVWMH score of 3; 1 point for a CS-EPVS or BG-EPVS score of 2 or higher; 1 point for the presence of CMB; and 1 point for the presence of a lacune. The sum of these four items yielded the total CSVD burden score, ranging from 0 to 4 points, with higher scores indicating a more severe burden of CSVD. For research purposes, we further categorized the CSVD burden into three grades: Grade 0 (0 points), Grade 1 (1 point), and Grade 2 (2–4 points).

### 2.3. BMD Measurement

Dual-energy X-ray Absorptiometry (DXA) is the gold standard for diagnosing osteoporosis. We used a DXA bone densitometer (Lunar Prodigy, GE Healthcare, Madison, WI, USA) to measure BMD at the lumbar spine (L1–L4) and the hip. The diagnosis was based on the corresponding T-scores: normal bone mass was defined as a T-score of −1.0 or higher; osteopenia was defined as a T-score between −1.0 and −2.5; and osteoporosis was defined as a T-score of −2.5 or lower. A patient was diagnosed with osteoporosis if the BMD at either the lumbar spine or the hip met the criteria for osteoporosis. If both sites showed normal BMD, the patient was classified in the normal group. Patients who did not meet the criteria for normal BMD or osteoporosis were classified in the osteopenia group.

### 2.4. Statistical Analysis

SPSS 21.0 statistical software was used for data analysis. Continuous variables following a normal distribution were expressed as mean ± standard deviation (SD) and compared among groups using independent-sample t-tests or analysis of variance (ANOVA). Non-normally distributed continuous variables were expressed as the median and interquartile range (IQR), with group comparisons made using non-parametric tests. Categorical variables were expressed as frequencies and percentages, and comparisons among groups were conducted using the chi-square test.

The inter-rater reliability of CSVD imaging marker assessments was evaluated using Cohen’s kappa statistic, with values greater than 0.8 indicating a high level of agreement. Ordered and binary logistic regression models were utilized to assess the relationship between BMD, osteoporosis, and both the overall CSVD burden and individual CSVD imaging features. In the ordered logistic regression model, common odds ratios (ORs) and 95% confidence intervals (CIs) were estimated using two models. Model 1 was unadjusted for covariates. In Model 2, adjustments were made for variables showing intergroup differences in univariate analyses, including age, SBP, TP, LDL-C, TG, FPG, ALT, eGFR, WBC count, RBC count, CRP, Fbg, gender, hypertension, PAD, AF, and CHD. Despite the absence of significant intergroup differences in diabetes, it was included due to its well-known impact on vascular health. Binary variables (PAD, AF, CHD, and diabetes) were coded as 1 = yes, and 0 = no. Osteoporosis status was reverse-coded (0 = osteoporosis, 1 = osteopenia, and 2 = normal) to allow for a more intuitive interpretation of the associations. To ensure the validity of the multivariable models, multicollinearity was assessed using variance inflation factors (VIFs). All included variables had VIF values less than 5, indicating no significant collinearity. For other CSVD imaging features, binary or multinomial logistic regression analyses were performed, with the OR and 95%CI reported. Similarly, Model 1 remained unadjusted for covariates, while Model 2 was adjusted for variables that exhibited intergroup differences in univariate analyses.

## 3. Results

This study included a total of 684 participants. The inter-rater reliability for CSVD imaging markers was high, with Cohen’s kappa values for brain atrophy, estimated lacunes, periventricular WMH, deep WMH, EPVS, and CMBs being 0.931, 0.914, 0.949, 0.940, 0.959, and 0.832, respectively, indicating strong agreement. The CSVD burden scores were distributed as follows: 257 participants with a score of 0 (CSVD = 0 group), 230 with a score of 1 (CSVD = 1 group), and 197 with scores of 2 or 3 (CSVD = 2 group). Among these participants, 90 had normal bone density, 288 had osteopenia, and 306 were diagnosed with osteoporosis. The number of hip osteoporosis cases was 239, and vertebral osteoporosis cases totaled 216. The participants had an average age of 68.4 years (SD = 10.3), with 222 males (32.5%) and 462 females (67.5%).

Table 1 compared the clinical characteristics and laboratory indicators across groups based on total CSVD burden scores. An increase in the total CSVD burden score was significantly associated with higher levels of age, SBP, FPG, WBC count, CRP, Fbg, and DD (*p* < 0.05). Conversely, TP, Alb, ALT, TC, HDL-C, LDL-C, eGFR, and RBC count decreased significantly (*p* < 0.05). As the total CSVD burden score increased, the proportions of osteoporosis, hip osteoporosis, and vertebral osteoporosis rose, while the proportions of normal bone mass and osteopenia in the hip and vertebrae decreased. Additionally, a heavier CSVD burden was associated with lower BMD values in both the hip and vertebrae (*p* < 0.05). TG levels also varied significantly across the three groups. Moreover, the proportions of patients with hypertension, PAD, AF, and CHD were higher in the groups with a greater CSVD burden score (*p* < 0.05).

On multivariable ordinal logistic regression analysis (Table 2), osteoporosis (*β* = 0.847, OR = 2.332, 95%CI: [1.345, 4.039], *p* = 0.003), hip osteopenia (*β* = 0.690, OR = 1.787, 95%CI: [1.125, 2.806], *p* = 0.014), hip osteoporosis (*β* = 0.955, OR = 2.598, 95%CI: [1.540, 4.384], *p* < 0.001), and vertebral osteoporosis (*β* = 0.416, OR = 1.515, 95%CI: [1.010, 2.272], *p* = 0.044) were significantly and independently associated with a greater CSVD burden (reference: CSVD = 0). Higher BMD at the hip (*β* = −0.073, OR = 0.929, 95%CI: [0.887, 0.972], *p* = 0.001) and at the vertebrae (*β* = −0.049, OR = 0.952, 95%CI: [0.917, 0.989], *p* = 0.012) was associated with lower odds of increased CSVD burden.

In sex-stratified analyses (Table 3), both hip and vertebral osteoporosis were independently associated with a higher CSVD burden in female patients (hip: OR = 2.529, 95%CI: [1.122, 5.703], *p* = 0.025; spine: OR = 3.129, 95%CI: [1.517, 6.455], *p* = 0.002; general osteoporosis: OR = 1.755, 95%CI: [1.057, 2.912], *p* = 0.03), and increased hip and vertebral BMD were correlated with lower odds of increased CSVD burden (OR = 0.907, 95%CI: [0.854, 0.963], *p* = 0.001; OR = 0.944, 95%CI: [0.899, 0.993], *p* = 0.025, respectively). In contrast, no significant association was observed in male patients (hip: OR = 1.964, 95%CI: [0.801, 4.816], *p* = 0.140; spine: OR = 1.625, 95%CI: [0.732,3.603], *p* = 0.232; general osteoporosis: OR = 2.332, 95%CI: [0.981, 5.540], *p* = 0.055), and changes in hip and vertebral BMD were not significantly associated with CSVD burden (OR = 0.973, 95%CI: [0.903, 1.050], *p* = 0.491; OR = 0.959, 95%CI: [0.901, 1.021], *p* = 0.197, respectively).

We performed logistic regression analyses on various imaging markers of CSVD, including lacunes, EPVS, WMH, and BA. As shown in Table 4, the results indicated that hip osteoporosis was an independent risk factor for lacunes (*β* = 0.795, OR = 2.215, 95%CI: [1.197, 4.1], *p* = 0.011), multiple lacunes (*β* = 0.822, OR = 2.274, 95%CI: [1.039, 4.98], *p* = 0.04), and severe WMH (*β* = 0.96, OR = 2.611, 95%CI: [1.171, 5.823], *p* = 0.019). Osteoporosis was independently associated with grade 2–4 EPVS (*β* = 0.799, OR = 2.222, 95%CI: [1.234, 4.004], *p* = 0.008). Additionally, hip osteopenia, hip osteoporosis, vertebral osteopenia, and vertebral osteoporosis were each independently associated with grade 2–4 EPVS (*β* = 0.551, OR = 1.735, 95%CI: [1.058, 2.844], *p* = 0.029; *β* = 0.688, OR = 1.99, 95%CI: [1.133, 3.495], *p* = 0.017; *β* = 0.415, OR = 1.514, 95%CI: [1.004, 2.284], *p* = 0.048; *β* = 0.502, OR = 1.652, 95%CI: [1.075, 2.538], *p* = 0.022). However, no significant association was found between BMD and BA (*p* > 0.05).

## 4. Discussion

Our study examined the relationship between osteoporosis and the burden of CSVD, including various imaging features of CSVD. While BA was analyzed, it was not found to be associated with osteoporosis. The findings underscore a significant association between osteoporosis and the overall CSVD burden, suggesting that bone health may influence cerebrovascular integrity. Subgroup analysis further revealed that this association was significant in female patients but not in males, indicating a potential sex-specific relationship. Given the observational nature of this study, causal inference is limited, and further longitudinal and interventional studies are needed to determine whether managing osteoporosis can causally impact CSVD risk.

### 4.1. Association Between Osteoporosis and CSVD Burden

The positive correlation between osteoporosis and increased CSVD burden observed in this study aligns with previous research suggesting that BMD and cerebrovascular health are interconnected [16,20]. Osteoporosis, characterized by reduced bone mass and microarchitectural deterioration, may reflect broader systemic processes that also affect cerebral vasculature. The pathophysiological mechanisms underlying this association may involve shared risk factors [25,26,27] such as aging, diabetes [27], and chronic inflammation, which can simultaneously contribute to bone loss and cerebrovascular damage [2]. Additionally, reduced BMD may indicate impaired calcium homeostasis and vitamin D deficiency, both of which have been implicated in endothelial dysfunction and increased vascular stiffness [28,29], leading to CSVD.

Our study found that even after adjusting for factors such as age, hypertension, and diabetes, the impact of osteoporosis on CSVD remained significant. This suggests that osteoporosis may influence cerebral small vessels through additional mechanisms. The underlying mechanisms of the association between osteoporosis and CSVD are not well elucidated; we propose that the influence of osteoporosis on CSVD primarily involves “bone–vascular crosstalk” and “bone–brain interaction”.

Firstly, the skeleton acts as an atypical endocrine organ. Osteocytes secrete various regulatory factors, such as sclerostin and osteoprotegerin (OPG), which play crucial roles in the interaction between bone, vasculature, and the brain. Sclerostin may disrupt the Wnt/β-catenin pathway in the brain, potentially contributing to aging and the progression of Alzheimer’s disease [11]. OPG, released by osteoblasts to inhibit bone resorption, also plays a significant role in the development of vascular wall inflammation and endothelial dysfunction [30]. Elevated OPG levels are associated with an increased risk of cardiovascular events [31]. Additionally, studies suggest that other proteins secreted by osteocytes, such as lipocalin-2 and fibroblast growth factor 23, may influence neuroinflammation, synaptic plasticity, and neuronal degeneration [32]. Aging-related bone factors may promote the development of atherosclerosis by affecting vascular smooth muscle cells [33]. These findings further reveal the link between osteoporosis, the central nervous system, and vascular diseases.

Furthermore, as a key site for stem cell maintenance and hematopoiesis, bone marrow provides endothelial progenitor cells (EPCs) and inflammatory cells. Research has found that aging-related extracellular vesicles (AB-EVs) from the bone matrix can infiltrate vascular tissues, promoting a bone–fat imbalance and vascular calcification [34]. We hypothesize that osteoporosis may disrupt the bone marrow microenvironment, thereby affecting stem cells critical for vascular repair and contributing to the progression of CSVD.

Finally, a recent study revealed that a loss-of-function mutation in the ARHGEF15 gene may inhibit the Wnt/β-catenin signaling pathway in osteoblasts, leading to dysfunction in vascular smooth muscle cells and osteoblasts [35]. This dysfunction manifests as CSVD accompanied by osteoporotic fractures, suggesting a potential shared genetic basis between osteoporosis and CSVD.

Importantly, our sex-stratified analysis revealed that the association between osteoporosis and CSVD burden was significant in female patients but not in males. This sex-specific disparity may be explained by the pivotal role of estrogen in maintaining both bone and vascular health [36]. In women, especially postmenopausal individuals, the abrupt decline in estrogen—a hormone known for its vasoprotective and neuroprotective properties [37]—can accelerate bone resorption while simultaneously promoting endothelial dysfunction [38], microvascular inflammation [39], and increased arterial stiffness. These processes may contribute to heightened susceptibility to CSVD. In contrast, men experience a more gradual decline in sex hormones, which may result in a lesser impact on the bone–vascular axis. Additionally, differences in body composition [40] and physical activity patterns, as well as the potential underdiagnosis of osteoporosis in men [41], may contribute to the observed disparities and warrant further investigation.

These findings indicate that it may be important to consider sex as a biological variable when studying the bone–brain axis, and suggest that postmenopausal women with osteoporosis could represent a particularly vulnerable group for CSVD-related brain changes. Future research should aim to elucidate the role of estrogen signaling in this interaction and assess whether sex-specific therapeutic strategies can mitigate cerebrovascular risk in osteoporotic populations.

### 4.2. Osteoporosis and Specific CSVD Imaging Markers

Our logistic regression analysis further revealed that hip osteoporosis is an independent risk factor for the presence of a lacune, multiple lacunes, and severe WMH. This is particularly noteworthy as lacunes and WMH are well-established markers of CSVD, associated with cognitive decline and an increased stroke risk [42,43,44]. The protective role of increased hip BMD against lacunes and severe WMH emphasizes the importance of maintaining bone health to potentially mitigate CSVD progression.

Additionally, the dose–response relationship observed between osteoporosis (including hip and vertebral osteoporosis) and higher grades of EPVS suggests that reduced bone density may contribute to the pathological expansion of EPVS. This relationship highlights the multifaceted nature of CSVD, where different imaging markers may be influenced by bone health through distinct mechanisms.

Interestingly, no significant association was found between BMD and brain atrophy in our study, diverging from previous reports [17,18,19] suggesting a link between osteoporosis and cortical thinning or brain volume reduction. This discrepancy may be due to differences in study populations or diagnostic criteria. Additionally, brain atrophy may lack specificity as a marker of CSVD, given that it can result from a range of other conditions. Beyond neurodegenerative disorders such as Alzheimer’s disease and chronic alcohol use, contributing factors include vitamin B12 deficiency, which has been linked to neurodegeneration and reduced brain volume. Moreover, age-related physiological brain volume loss further complicates the interpretation of its relationship with CSVD. However, the association between osteoporosis and the overall CSVD burden suggests that bone health may exert a cumulative effect on multiple CSVD subtypes. Further research is needed to clarify the relationship between osteoporosis and BA, especially in diverse cohorts and using more sensitive imaging modalities.

The notably low incidence of CMBs observed in our study differs from previous research. This could be due to our study population, which included non-stroke individuals, some from general health check-up groups. Consequently, the risk of hemorrhagic transformation was likely lower, and antiplatelet medication use was less common, contributing to the reduced CMB occurrence.

Despite this study’s merits, such as thorough imaging evaluations and meticulous clinical and laboratory data collection, several limitations should be acknowledged. The retrospective, hospital-based design may limit the generalizability of our findings to the broader population, as selection bias cannot be excluded. Additionally, the modest sample size constrains the statistical power and external validity, underscoring the need for multicenter validations. The cross-sectional nature of this study precludes causal inferences, making it unclear whether osteoporosis contributes to increased CSVD burden or vice versa. Residual confounding from unmeasured variables also cannot be ruled out. Moreover, we did not exclude individuals with a history of fractures, which may have influenced bone mineral density independently of osteoporosis and introduced additional variability. Furthermore, the exclusion of bone-related biomarkers, such as vitamin D and osteocalcin, due to incomplete data, hampers a deeper understanding of the mechanisms linking osteoporosis with cerebrovascular disease. Additionally, some odds ratios had relatively wide confidence intervals, suggesting potential estimate variability due to the modest sample size or population heterogeneity. Future studies with larger, more diverse cohorts and refined analyses are needed to validate these findings and enhance estimate precision.

These findings carry important clinical implications, suggesting that osteoporosis management may play a role in preventing or slowing the progression of CSVD. With the increasing recognition of CSVD as a major contributor to cognitive decline and stroke, interventions aimed at improving bone health—such as calcium and vitamin D supplementation, weight-bearing exercises, and pharmacological treatments for osteoporosis—could have the added benefit of preserving both bone and cerebrovascular health.

## 5. Conclusions

Our study demonstrates a significant association between osteoporosis and an increased CSVD burden, including specific CSVD imaging markers, with the exception of BA. This association with total CSVD burden was particularly evident in female patients, indicating a potential sex-related difference. These results suggest a potential link between osteoporosis and increased CSVD burden, particularly in women, warranting further investigation into the underlying mechanisms.

## Figures and Tables

**Table 1:** Demographic and clinical characteristics according to the CSVD burden.

	CSVD = 0 (257)	CSVD = 1 (230)	CSVD = 2 ^a^ (197)	*p*	*F/H/χ* ^2 e^
Age (year, median (IQR))	61 (56, 69)	69 (61, 75)	76 (69, 82)	<0.001	158.9
SBP (mmHg, median (IQR))	127 (115, 142)	133 (121, 145)	138 (125, 152)	<0.001	29.3
BMI (kg/m^2^, median (IQR))	23.61 (21.91, 24.83)	23.44 (21.64, 25.64)	23.56 (21.57, 24.95)	0.713	0.7
TP (g/L, median (IQR))	70.8 (67.4, 74.7)	70.55 (66.8, 74.5)	68.6 (64.8, 72.5)	<0.001	20.5
Alb (g/L, median (IQR))	41.6 (39.2, 43.7)	40.56 (38, 42.9)	38.6 (36.1, 41.1)	<0.001	55.9
ALT (U/L, median (IQR))	18.3 (13, 26.3)	15.9 (12, 22.6)	15.8 (12, 20.8)	<0.001	16.4
AST (U/L, median (IQR))	20 (17.3, 26.7)	19.4 (16.6, 23.5)	20.2 (17, 24.8)	0.194	3.3
ALP (U/L, median (IQR))	77.2 (63, 92.2)	77.95 (62.1, 94.5)	79.7 (63.7, 93)	0.870	0.3
TC (mmol/L, median (IQR))	5.04 (4.23, 5.91)	4.89 (4.2, 5.66)	4.56 (3.88, 5.18)	<0.001	24.3
HDL-C (mmol/L, median (IQR))	1.34 (1.12, 1.51)	1.27 (1.08, 1.5)	1.21 (1.01, 1.45)	0.005	10.8
LDL-C (mmol/L, median (IQR))	3.34 (2.69, 3.99)	3.23 (2.67, 3.79)	2.9 (2.39, 3.52)	<0.001	21.2
TG (mmol/L, median (IQR))	1.28 (0.97, 1.81)	1.47 (1.06, 1.97)	1.23 (0.9, 1.76)	0.008	9.7
FPG (mmol/L, median (IQR))	5.47 (4.97, 6.55)	5.74 (5.09, 6.9)	5.75 (5.02, 6.89)	0.090	4.8
eGFR(mL/min/1.73 × m^2^, median (IQR))	94.7 (86.3, 102.3)	89.6 (74.8, 96.5)	84.2 (72.3, 91.9)	<0.001	72.3
UA (umol/L, median (IQR))	334 (272.2, 394)	347.9 (276.5, 404.9)	328.1 (275, 403)	0.492	1.4
WBC (×10^9^/L, median (IQR))	5.94 (5.09, 6.05)	6.10 (5.14, 7.33)	6.57 (5.61, 7.91)	<0.001	16.7
PLT (×10^9^/L, median (IQR))	229 (192, 262)	222 (189, 265)	228 (189, 264)	0.732	0.6
RBC (×10^12^/L, median (IQR))	4.38 (4.09, 4.73)	4.31 (3.94, 4.66)	4.17 (3.77, 4.52)	<0.001	18.7
CRP (mg/L, median (IQR))	2 (0.88, 4.36)	2.39 (0.97, 7)	3 (1.22, 7.9)	0.007	9.8
DD (ug/mL, median (IQR))	0.36 (0.23, 0.87)	0.51 (0.26, 1.14)	0.76 (0.37, 1.35)	<0.001	9.2
Fbg (g/L, median (IQR))	3.06 (2.64, 3.49)	3.15 (2.72, 3.6)	3.26 (2.78, 3.82)	0.010	39.0
Female, *n* (%)	184 (71.6)	156 (67.8)	122 (61.9)	0.092	4.8
Diabetes, *n* (%)	92 (35.8)	91 (39.6)	90 (45.7)	0.102	4.6
Hypertension, *n* (%)	104 (40.5)	125 (54.3)	148 (75.1)	<0.001	54.2
Dyslipidemia, *n* (%)	108 (42.4)	98 (42.6)	67 (34.0)	0.121	4.2
PAD, *n* (%)	92 (35.8)	110 (47.8)	120 (60.9)	<0.001	28.3
Hyperuricemia, *n* (%)	42 (16.7)	45 (19.6)	38 (19.3)	0.787	0.7
AF, *n* (%)	1 (0.40)	6 (2.60)	7 (3.60)	0.033	6.8
CHD, *n* (%)	13 (5.10)	19 (8.30)	31 (15.7)	<0.001	15.6
Current smoking, *n* (%)	21 (8.20)	17 (7.40)	15 (7.60)	0.946	0.1
Current dinking, *n* (%)	16 (6.60)	13 (5.70)	10 (5.10)	0.777	0.5
Spine BMD (g/cm^2^, median (IQR))	1.007 (0.868, 1.126)	0.937 (0.826, 1.807)	0.926 (0.778, 1.098)	0.002	12.4
Hip BMD (g/cm^2^, mean ± SD)	0.856 ± 0.143	0.799 ± 0.163	0.758 ± 0.174	<0.001	21.5
Normal ^b^, *n* (%)	49 (19.1)	27 (11.7)	14 (7.10)	<0.001	40.1
Osteopenia ^c^, *n* (%)	126 (49.4)	96 (41.7)	65 (33.0)		
Osteoporosis ^d^, *n* (%)	80 (31.5)	107 (46.5)	118 (59.9)		
Normal (hip), *n* (%)	65 (25.3)	33 (14.3)	16 (8.10)	<0.001	55.5
Osteopenia (hip), *n* (%)	138 (53.7)	114 (49.6)	79 (40.1)		
Osteoporosis (hip), *n* (%)	53 (21.0)	83 (36.1)	102 (51.8)		
Normal (spine), *n* (%)	101 (39.3)	76 (33.0)	64 (32.5)	0.007	14.0
Osteopenia (spine), *n* (%)	92 (35.8)	82 (35.7)	53 (26.9)		
Osteoporosis (spine), *n* (%)	63 (24.9)	72 (31.3)	80 (40.6)		

SBP systolic blood pressure, BMI body mass index, TP total protein, Alb albumin, ALT alanine aminotransferase, AST aspartate aminotransferase, ALP alkaline phosphatase, TC total cholesterol, HDL-C high-density lipoprotein cholesterol, LDL-C low-density lipoprotein cholesterol, TGs triglycerides, FPG fasting plasma glucose, eGFR estimated glomerular filtration rate, UA uric acid, *WBC* white blood cell, RBC red blood cell, CRP C-reactive protein, DD D dimer, Fbg fibrinogen, PAD peripheral artery disease, AF atrial fibrillation, CHD coronary heart disease, BMD bone mineral density. ^a^ Defined as a CSVD burden score of 2 or 3; ^b^ defined as both spine and hip showing normal BMD; ^c^ defined as any site meeting criteria for osteopenia but not yet reaching osteoporosis; ^d^ diagnosed if the BMD at either the lumbar spine or the hip met the criteria for osteoporosis; ^e^ “*F*” represents the F-statistic from one-way ANOVA, “*H*” denotes the H-statistic from the Kruskal–Wallis test, and “χ^2^” corresponds to the chi-square test for categorical variables.

**Table 2:** Association between osteoporosis and CSVD burden.

Variables	Model 1		Model 2	
	Unadjusted OR (95%CI)	*p*	Adjusted OR (95%CI)	*p*
Osteopenia ^a^	1.532 (0.974, 2.410)	0.065	1.535 (0.924, 2.552)	0.097
Osteoporosis ^b^	3.333 (2.119, 5.243)	<0.001 *	2.332 (1.345, 4.039)	0.003 *
Hip osteopenia	1.871 (1.241, 2.826)	0.003 *	1.787 (1.125, 2.806)	0.014 *
Hip osteoporosis	4.517 (2.921, 6.986)	<0.001 *	2.598 (1.540, 4.384)	<0.001 *
Spine osteopenia	0.971 (0.694, 1.359)	0.866	1.225 (0.834, 1.796)	0.300
Spine osteoporosis	1.69 (1.203, 2.375)	0.002 *	1.515 (1.01, 2.272)	0.044 *
Hip BMD/(2 × LSD ^c^)	0.886 (0.853, 0.920)	<0.001 *	0.929 (0.887, 0.972)	0.001 *
Spine BMD/(2 × LSD ^d^)	0.953 (0.924, 0.982)	0.002 *	0.952 (0.917, 0.989)	0.012 *

BMD bone mineral density, LSD the least significant change. ^a^ Defined as any site meeting the criteria for osteopenia but not yet reaching osteoporosis; ^b^ diagnosed if the BMD at either the lumbar spine or the hip met the criteria for osteoporosis; ^c^ the value is 0.021 g/cm^2^ in our hospital; ^d^ the value is 0.024 g/cm^2^ in our hospital. * *p* < 0.05.

**Table 3:** Sex-stratified analysis of the association between osteoporosis and CSVD burden.

Variables	Male		Female	
	OR (95%CI)	*p*	OR (95%CI)	*p*
Osteopenia ^a^	1.52 (0.756, 3.055)	0.239	1.724 (0.771, 3.853)	0.184
Osteoporosis ^b^	2.332 (0.981, 5.540)	0.055	2.529 (1.122, 5.703)	0.025 *
Hip osteopenia	1.938 (0.999, 3.765)	0.050	1.993 (1.003, 3.962)	0.049 *
Hip osteoporosis	1.964 (0.801, 4.816)	0.140	3.129 (1.517, 6.455)	0.002 *
Spine osteopenia	0.761(0.392, 1.476)	0.420	1.558 (0.954, 2.549)	0.077
Spine osteoporosis	1.625 (0.732, 3.603)	0.232	1.755 (1.057, 2.912)	0.030 *
Hip BMD/(2 × LSD ^c^)	0.973 (0.903, 1.050)	0.491	0.907 (0.854, 0.963)	0.001 *
Spine BMD/(2 × LSD ^d^)	0.959 (0.901, 1.021)	0.197	0.944 (0.899, 0.993)	0.025 *

*BMD* bone mineral density, *LSD* the least significant change. ^a^ Defined as any site meeting the criteria for osteopenia but not yet reaching osteoporosis; ^b^ diagnosed if the BMD at either the lumbar spine or the hip met the criteria for osteoporosis; ^c^ the value is 0.021 g/cm^2^ in our hospital; ^d^ the value is 0.024 g/cm^2^ in our hospital. * *p* < 0.05.

**Table 4:** Association between osteoporosis and CSVD imaging features.

Imaging Features	Variables	Model 1		Model 2	
		Unadjusted OR (95%CI)	*p*	Adjusted OR (95%CI)	*p*
BA = 1 ^a^	Osteopenia ^b^	0.923 (0.574, 1.482)	0.740	0.608 (0.339, 1.090)	0.095
	Osteoporosis ^c^	2.533 (1.562, 4.116)	<0.001 *	1.030 (0.543, 1.952)	0.928
	Hip osteopenia	1.243 (0.79, 1.956)	0.346	0.726 (0.376, 1.400)	0.339
	Hip osteoporosis	5.359 (2.97, 9.671)	<0.001 *	2.141 (0.906, 5.060)	0.083
	Spine osteopenia	0.903 (0.6, 1.36)	0.626	0.961 (0.516, 1.79)	0.899
	Spine osteoporosis	1.8 (1.139, 2.843)	0.012 *	1.019 (0.496, 2.095)	0.959
BA = 2 ^a^	Osteopenia ^b^	0.923 (0.574, 1.482)	0.740	0.608 (0.339, 1.090)	0.095
	Osteoporosis ^c^	2.533 (1.562, 4.116)	<0.001 *	1.030 (0.543, 1.952)	0.928
	Hip osteopenia	10.207 (4.238, 24.582)	0.817	0.439 (0.136, 1.409)	0.166
	Hip osteoporosis	0.904 (0.384, 2.219)	<0.001 *	2.411 (0.642, 9.050)	0.192
	Spine osteopenia	0.507 (0.697, 2.551)	0.045	0.822 (0.309, 2.185)	0.694
	Spine osteoporosis	1.333 (0.697, 2.551)	0.385	0.731 (0.261, 2.043)	0.550
Lacune	Osteopenia ^b^	1.354 (0.798, 2.295)	0.261	1.292 (0.682, 2.449)	0.432
	Osteoporosis ^c^	2.86 (1.703, −4.802)	<0.001 *	1.773 (0.924, 3.403)	0.085
	Hip osteopenia	1.626 (0.998, 2.649)	0.051	1.492 (0.838, 2.659)	0.174
	Hip osteoporosis	4.328 (2.619, 7.151)	<0.001 *	2.215 (1.197, 4.100)	0.011 *
	Spine osteopenia	0.718 (0.492, 1.048)	0.086	0.823 (0.523, 1.297)	0.402
	Spine osteoporosis	1.378 (0.951, 1.997)	0.090	0.622 (0.26, 1.485)	0.419
Multiple lacunes ^d^	Osteopenia ^b^	1.521 (0.733, 3.513)	0.013 *	1.606 (0.715, 3.606)	0.251
	Osteoporosis ^c^	2.462 (1.212, 4.999)	0.010 *	1.73 (0.763, 3.921)	0.189
	Hip osteopenia	1.55 (0.774, 3.102)	0.216	1.497 (0.701, 3.198)	0.297
	Hip osteoporosis	3.878 (1.962, 7.665)	<0.001 *	2.274 (1.039, 4.98)	0.04 *
	Spine osteopenia	0.852 (0.531, 1.369)	0.509	1.087 (0.637, 1.856)	0.76
	Spine osteoporosis	1.084 (0.684, 1.72)	0.731	1.025 (0.603, 1.743)	0.926
EPVS ≥ 2 ^e^	Osteopenia ^b^	1.499 (0.896, 2.507)	0.123	1.673 (0.967, 2.896)	0.066
	Osteoporosis ^c^	2.131 (1.282, 3.543)	0.004 *	2.222 (1.234, 4.004)	0.008
	Hip osteopenia	1.628 (1.027, 2.581)	0.038 *	1.735 (1.058, 2.844)	0.029 *
	Hip osteoporosis	2.165 (1.342, 3.491)	0.002 *	1.99 (1.133, 3.495)	0.017 *
	Spine osteopenia	1.184 (0.815, 1.721)	0.375	1.514 (1.004, 2.284)	0.048 *
	Spine osteoporosis	1.498 (1.029, 2.18)	0.035	1.652 (1.075, 2.538)	0.022 *
WMH	Osteopenia ^b^	1.34 (0.812, 2.211)	0.252	1.209 (0.676, 2.162)	0.523
	Osteoporosis ^c^	2.759 (1.63, 4.668)	<0.001 *	1.608 (0.849, 3.047)	0.145
	Hip osteopenia	1.449 (0.925, 2.27)	0.105	1.164 (0.694, 1.952)	0.566
	Hip osteoporosis	4.064 (2.369, 6.971)	<0.001 *	1.837 (0.974, 3.467)	0.060 *
	Spine osteopenia	0.882 (0.587, 1.325)	0.545	1.052 (0.656, 1.685)	0.834
	Spine osteoporosis	1.606 (1.024, 2.52)	0.039 *	1.336 (0.784, 2.276)	0.287
Severe WMH ^f^	Osteopenia ^b^	1.6 (0.774, 3.309)	0.205	1.649 (0.716, 3.794)	0.240
	Osteoporosis ^c^	3.386 (1.679, 6.83)	0.001 *	2.041 (0.901, 4.622)	0.087
	Hip osteopenia	2.21 (1.088, 4.486)	0.028 *	2.061 (0.931, 4.562)	0.075
	Hip osteoporosis	5.332 (2.642, 10.758)	<0.001 *	2.611 (1.171, 5.823)	0.019 *
	Spine osteopenia	0.842 (0.532, 1.333)	0.464	1.085 (0.633, 1.859)	0.766
	Spine osteoporosis	1.402 (0.909, 2.162)	0.126	1.193 (0.717, 1.987)	0.497

BMD bone mineral density, BA brain atrophy, EPVSs enlarged perivascular spaces, WMHs white matter hyperintensities. ^a^ Ordinal logistic regression was used with BA = 0 as the control group for the independent variables osteopenia and osteoporosis, while multinomial logistic regression was applied to other variables; ^b^ defined as any site meeting the criteria for osteopenia but not yet reaching osteoporosis; ^c^ diagnosed if the BMD at either the lumbar spine or the hip met the criteria for osteoporosis; ^d^ defined as three or more lacunes present; ^e^. defined as more than 10 EPVSs in the basal ganglia and centrum semiovale; ^f^. defined as a deep white matter hyperintensity (WMH) Fazekas score of 2 and/or a periventricular WMH score of 3. * *p* < 0.05.

## Data Availability

The datasets analyzed in this study are available from the corresponding author upon reasonable request.

## Abbreviations

The following abbreviations are used in this manuscript:

CSVD	Cerebral Small Vessel Disease
EPVSs	Enlarged Perivascular Spaces
WMHs	White Matter Hyperintensities
BA	Brain Atrophy
BMD	Bone Mineral Density
CHD	Coronary Heart Disease
AF	Atrial Fibrillation
PAD	Peripheral Artery Disease
BMI	Body Mass Index
SBP	Systolic Blood Pressure
DBP	Diastolic Blood Pressure
WBC	White Blood Cell Count
RBC	Red Blood Cell Count
Hb	Hemoglobin
LDL-C	Low-Density Lipoprotein Cholesterol
TGs	Triglycerides
TC	Total Cholesterol
FPG	Fasting Plasma Glucose
ALT	Alanine Aminotransferase
eGFR	Estimated Glomerular Filtration Rate
CRP	C-Reactive Protein
DD	D-Dimer
TP	Total Protein
Alb	Albumin
Fbg	Fibrinogen
AST	Aspartate Aminotransferase
ALP	Alkaline Phosphatase
HDL-C	High-Density Lipoprotein Cholesterol
UA	Uric Acid
